# A colossal atrial myxoma

**DOI:** 10.4103/0975-3583.70923

**Published:** 2010

**Authors:** Atoosheh Rohani, Vahid Akbari

**Affiliations:** *Yasuj University of Medical Science, Yasuj, Iran*; 1 *Sajjad Hospital, Yasuj, Iran*

**Keywords:** Hypesthesia, myxomas, vertigo

## Abstract

Atrial myxomas are the most common benign primary tumor of the heart. These cardiac growths can masquerade as mitral stenosis and infective endocarditis. A 35-year-old man presented with complaints of nonspecific symptoms. Echocardiogram revealed a large atrial myxoma occupying the left atrium. Excision revealed a 14 × 8 × 6 cm3 tumor attached to a 4 × 3 × 2 cm3 stalk of septal tissue. We describe a giant left atrial myxoma. We were not able to find another myxoma as big as this one in the literature, so we are reporting it.

## INTRODUCTION

The first description of a primary intracardiac tumor was in 1559, located in the left ventricle.[1] Atrial myxomas are the most common benign primary tumor of the heart and occur in as many as 3 in 1000 patients.[2] Most cases of atrial myxoma are sporadic, and the exact etiology is unknown. Myxomas account for 40–50% of primary cardiac tumors. Approximately 90% are solitary and pedunculated, and 75–85% occurs in the left atrial cavity.[3,4] Because of nonspecific symptoms, early diagnosis may be a challenge. We present the case of a 35-year-old man with a large atrial myxoma, who presented with complaints of nonspecific symptoms and who was found to have a grade 2/6 systolic murmur.

## CASE REPORT

A 35-year-old man was referred to our cardiology clinic due to his nonspecific symptoms such as atypical chest pain, numbness in his left hand, palpitation, and dizziness (not positional). He did not complain of any constitutional symptoms. He did not have any previous medical problem; there was no history of peripheral or pulmonary embolization. The patient had no prior history of heart murmurs, syncope, and shortness of breath.

Findings on physical examination were: normal sinus rhythm, at the rate of 84 per minute and blood pressure 124/84mmHg. Jugular venous pressure was normal, and basilar pulmonary rales was heard. On heart auscultation, there was a loud S_1_, a grade 2/6 holosystolic high-pitched murmur at the apex with radiation to the left axilla. There was also an early diastolic sound, now we know it was tumor plop. The remainder of the physical examination was unremarkable.

Laboratory data included a normal hemogram, urinalysis, and electrolytes. Erythrocyte sedimentation rate (ESR) and C-reactive protein levels were elevated.

Liver function and coagulation tests were normal. Chest roentgenogram showed mild cardiomegaly, electrocardiogram was abnormal due to left axis deviation, left atrial enlargement, and PR interval was 240 ms.

**Figure 1:**
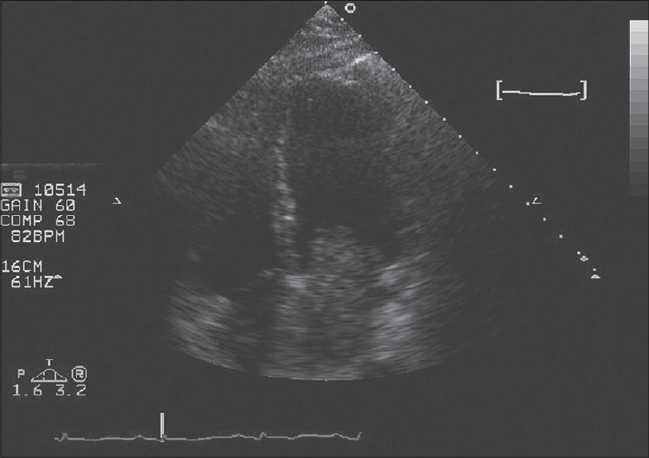
Large LA myxoma

**Figure 2:**
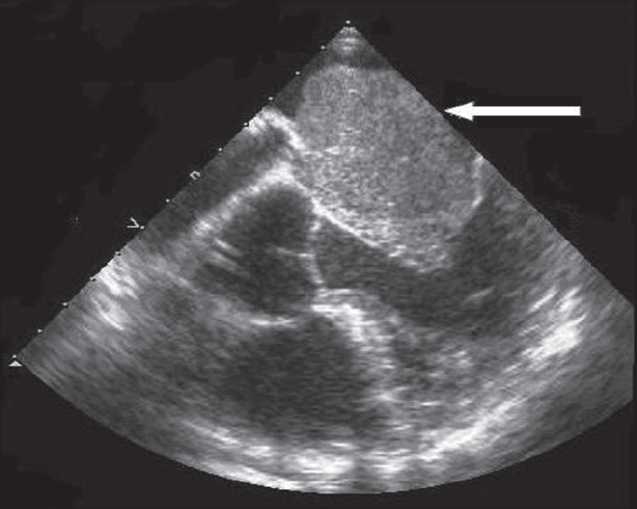
Transesophagial echo

Two-dimensional echocardiography was done and a huge mobile LA mass with a stalk that protrude in LV during systole was appeared, it caused severe eccentric jet of MR [Figure 1]. Then, patient underwent transesophageal echocardiography [Figure 2].

It revealed a huge mass was attached to interatrial septum and severe MR. Patient was referred to a cardiac surgeon. An emergency operation was performed after the diagnosis. The left atrium was incised along the interatrial groove to find a huge myxoma occupying most of the left atrium, with a part of it incarcerating into the left ventricle. Its stalk was attached to the inferior edge of the fossa ovalis. A part of the atrial septum, where the stalk of the myxoma was attached, was excised along with the huge myxoma. The atrial septal defect was closed with a pericardial patch. A yellowish gelatinous mass with a lobulated surface was removed. Prolapsed of a tumor through the mitral valve resulted in the destruction of the annulus. Mitral valve was repaired (by ring annuloplasty).

### Histologic findings

**Figure 3:**
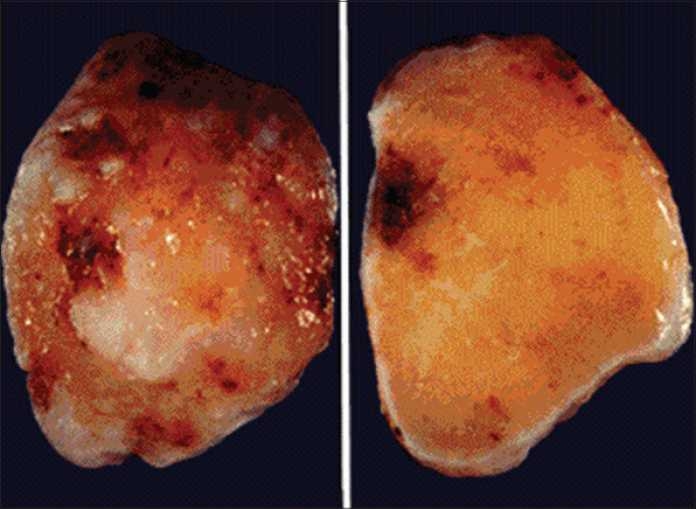
Yellowish mass excised

**Figure 4:**
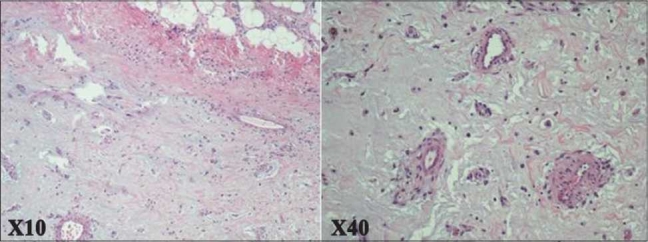
Histological section of atrial myxoma

Its size was 14 × 8 × 6 cm^3^and weight was 458 g [Figures 3 and 4]. Microscopic examination showed stellate-shaped in a vascular myxoid stroma. The cells are polygonal with scant eosinophilic cytoplasm.

Tumor necrosis is present. Immunohistochemical expression showed of interleukin-6, but had no abnormal DNA content.

## DISCUSSION

Prior to 1951, the diagnosis of intracardiac tumors was made only at postmortem examination; in that year the diagnosis of a left atrial tumor was confirmed by angiocardiography.[5] Chest radiograph may reveal left atrial enlargement and signs of pulmonary hypertension and congestion. Transthoracic two-dimensional echocardiography and if necessary the transesophageal approach can be used to determine the location, size, shape, point of attachment, and motion characteristics of a myxoma. The latter investigation is particularly helpful for precise delineation of tumor size, number, and attachments. Our patient failed to present with the common symptoms associated with atrial myxoma including dyspnea, orthopnea, peripheral embolism, or syncope. Above clinical manifestations can occur separately or simultaneously, and can be either symptomatic or asymptomatic depending on the extent of the tumor growth, and we think that our patient’s tumor must be slow growth because of patient was oligosymptomatic, with recent initiation of palpitation and mild precordialgia. In addition, an obstruction of the mitral valve outflow by the tumor is often related to the patient’s posture, and because our patient’s job was bus driver and he was in upright posture all of the time, he did not have dyspnea. In this study, we found a case that was difficult to diagnose, due to the small amount of signs and symptoms. The patient underwent surgery (without complications) and an anatomic-pathological study confirmed that it was myxoma.

## CONCLUSION

This patient did not present with the common symptoms associated with an atrial myxoma. Although atrial myxomas are usually benign or asymptomatic, there is the possibility of diastolic embolization,[2,3] conduction alterations and disturbances,[4] and lethal valve obstructions occurring.[6] Since surgical excision has been reported to alleviate symptoms associated with cardiac myxomas, early identification and removal are preferable. We recommend echocardiogram in the workup of atypical chest pain and vertigo of unknown etiology; we hope to facilitate earlier identification of these intracardiac growths.
